# Health problems during childhood and school achievement: Exploring associations between hospitalization exposures, gender, timing, and compulsory school grades

**DOI:** 10.1371/journal.pone.0208116

**Published:** 2018-12-05

**Authors:** Cristian Bortes, Mattias Strandh, Karina Nilsson

**Affiliations:** 1 Department of Social Work, Faculty of Social Sciences, Umeå University, Umeå, Sweden; 2 Centre for Research on Child and Adolescent Mental Health, Karlstad University, Karlstad, Sweden; 3 Department of Sociology, Faculty of Social Sciences, Umeå University, Umeå, Sweden; TNO, NETHERLANDS

## Abstract

**Aims:**

To investigate while accounting for health at birth 1) associations between health problems during childhood, measured as hospitalizations, and school achievement in the final year of compulsory school, measured as overall grade points and eligibility for upper secondary education, 2) if and how gender moderates the association between health problems and school achievement, 3) if and how the timing of a health problem during childhood is associated with later school achievement.

**Methods:**

Analyzes were performed on a population-based cohort (*n* = 115 196) born in 1990 in Sweden (51.3% boys, 48.7% girls) using data from several national registries. Multiple linear regression and logistic regression were used to analyze associations between study variables.

**Results:**

Overall grade points and eligibility for continuation to upper secondary school were lower for individuals exposed to hospitalizations. Only the association between hospitalizations and overall grade points was moderated by gender and only for ages 13–16 years. Exposure close to actual grading had worst outcomes.

**Conclusions:**

Health problems, measured through hospitalizations, was significantly associated with lower school achievements among Swedish children. Girls exposed to health problems requiring hospitalizations had relatively poorer school achievements as compared to boys. Health problems requiring hospitalization during junior high school had the greatest negative association with final achievement at compulsory school.

## Introduction

Many factors across multiple domains influence whether children and adolescents succeed in their educational attainment [1–2], and health is an important prerequisite for successful schooling [3–11]. Previous research on childhood health and later educational outcomes has largely been both condition specific and country specific. The World Health Organization has stated that the overwhelming majority of research is from the United States and has called for further European evidence [12]. As health and educational systems differ between countries, findings from one country might not be directly transferrable to others. In Sweden, research into how health affects education is categorized as a field “under development” [13], meaning that gaps in research still remain. Apart from one previous study [14], Swedish register data has been underutilized in investigating the relationship between health during childhood and school achievements and, furthermore, the study did not take into account birth health status, which has proven to be a significant predictor of childhood health and future life outcomes [4,5]. In the present study, we further utilized the potential of Swedish register data, including several indicators of pre-natal and birth health status, to investigate how health problems during childhood, measured as hospitalizations, are associated with school achievement in the form of overall grade points and eligibility for upper secondary education in the final year of compulsory school.

One central issue in research in education and schoolchildren’s health has long been gender differences. It has long been recognized that girls as a group perform better than boys in school [15], and also that girls in most of the industrialized countries have poorer self-reported health [16], which also is the case in Sweden [17]; girls in the early teens are more likely to report poor health and multiple health complaints compared with boys [18–20]. Although these differences are of great public health interest, they have only been studied separately; some have studied gender differences in school achievement on one hand [21,22] others gender differences in health on the other [23,24]. Consequently, there is currently a lack of systematic comparison and unlike previous investigations, the present study formally tested differences and examined whether girls and boys with health problems requiring hospitalization are affected differently in their school achievement.

Another important aspect we for reasons related to lack of sufficient data [7] have limited knowledge of, is if and how the timing of a health problem during childhood impacts later school achievement. Studies based on longitudinal data from the British National Child Development Study [25–28], albeit with a wider scope in studying childhood health and later life outcomes, have revealed a cumulative effect of so-called “systems” conditions (which include heart, lungs, digestive, blood, urogenital and neurological conditions) on educational outcomes, suggesting that the timing of when a health condition occurs is important. However, this was not studied further. Needless to say, these studies were limited regarding national and temporal contexts as they only focused on Great Britain and its educational system during the 1970’s. In a Swedish context, a register based study found that later hospitalizations had a greater impact on grades [15] while research into one specific health condition, i.e., diabetes, found that the condition is not only associated with lower school achievements [29–31] but the age of onset seems to play a role [32] and there is a cumulative effect of the disease as well: children with earlier onset diabetes suffer a greater disadvantage in their schooling, reflected as lower school grades, as a result of the disease [33]. Apart from these studies, the question of timing has received little attention.

### Study aims

The present study aimed to investigate the association between health problems requiring hospitalization during childhood and school achievements in compulsory school in the form of 1) overall grade points, and 2) eligibility for continuation to upper secondary education. Overall grade points indicate differences in levels of school achievement while eligibility for continuation to upper secondary education indicate problems of completing education at all. The study expands current knowledge in three ways. First, using hospitalizations as a measure of health problems is advantageous as it includes many different health conditions, it thus serves as a summarizing measure of health problems in the study-population, in contrast to previous mostly condition-specific research, while at the same time indicating serious health problems. Second, we specifically investigated if and how gender moderates the association between hospitalizations on school achievement measured as overall grade points and eligibility for upper secondary education. Third, we investigated the timing of hospitalization exposure; the differences between exposure to hospitalizations early in life before entrance into the Swedish school system vs. exposure to hospitalizations in later years after school entry, and its association with school achievements.

## Materials and methods

Data were provided by the Umeå SIMSAM Lab infrastructure [34], which was designed to address questions on childhood, health and welfare. It covers the entire Swedish population between 1960 and 2010, and includes micro-level information from a large number of registers. Individuals are linked between registers with a unique, anonymized, personal identification number. We used data from the Medical Birth Register (MBR) to obtain information on birth health status (for content, quality and previous use of the MBR see Källén and Källén [35], Axelsson [36] and Odlind et al. [37].). Information on hospitalizations and length of stay at medical care events was obtained from the Swedish National Patient Register (NPR). The NPR has been validated in previous studies [38,39]. Information on grades was obtained from the Swedish National Agency of Education’s Pupil Register. Data on parental education level and family type were obtained from Statistics Sweden. The present study total population comprised every person born in 1990 who was alive and residing in Sweden in 2010 (*n* = 135 027). Three exclusions were made, i.e., all foreign-born individuals, all persons born in Sweden with missing data on Apgar score 5 minutes after birth (because we wanted to include data on birth health status) and the few individuals with adoptive mothers and/or fathers. After these exclusions, our analytical sample consisted of *n* = 115 196 individuals (59 140 boys (51.3%) and 56 056 girls (48.7%)). The Regional Ethical Vetting Board in Umeå approved all research based on data from the Umeå SIMSAM Lab, including the present study.

### School achievement

School achievement was measured using two variables: *overall grade points* and *eligibility for upper secondary education*. Overall grade points is the sum of the 16 best subject grades in 9^th^ and final grade of compulsory school; it is received at age 15–16. The grading points indicate differences in levels of school achievement and are a summary of performance, with grades ranging from 0 to 320 points. For every subject, students are assigned a grade ranging from 0 to 20, where 0 is failure. The lowest score on all subjects is 0, implying that one has scored 0 in all the tested subjects, while the highest is 320, implying that one has scored 20 in all 16 subjects. Overall grade points is a continuous variable and fairly normally distributed In our study sample, 3238 individuals had missing grade points. These individuals had failed to complete compulsory school on time. We coded their overall grade points as “0”.

Eligibility for upper secondary education was assessed at the time the cohort was in the ninth grade based on having completed compulsory school with pass grades in the core subjects Swedish, English, and mathematics, which was a requirement for studies in national upper secondary level education programs. A dummy variable (0/1) was created for passing grades in all three core subjects (0) or failure in one or more of the three core subjects (1), thus assessing ineligibility for upper secondary education.

### Health problems

As a measure of health problems, we used *hospitalizations*. Information on hospitalizations was retrieved from the NPR and contains data on all in-patient medical care events in Swedish hospitals. If an individual had experienced a medical care event in a particular year, we defined that individual as exposed to health problems that year. Based on the total length of stay an individual could have a score of “0” in the registry, which indicated a visit to a hospital but without an overnight stay. We defined a score greater than “0” as a serious health problem because it required overnight hospitalization and less than “1” as no hospitalization. We used the number of nights hospitalized in our analyzes. Furthermore, since we were interested in the timing aspect of exposure to health problems, time of exposure was defined by having been hospitalized only during the years prior to formal schooling (age 0–6 years), only during the years after school entry through middle school (age 7–12 years), or only during the years in junior high school (age 13–16 years). These age range groupings were chosen due to the institutional schooling structure in Sweden.

### Covariates

*Gender* was dummy coded as male = 0, female = 1. *Apgar score at 5 minutes* is a measure of a new-born’s physical condition after birth (low/normal): an Apgar score lower than 7 is considered low and within 7–10 is normal [40]. Owing to the exclusions, there were no missing data for Apgar score. Data on *low or high birthweight* at gestational age [41] were also included (yes/no). Data were missing for 2.5% of the cohort. *Maternal smoking habits* upon admission to maternity care (no smoking, 1–9 cigarettes/day, ≥10 cigarettes/day) can be used as an indicator of in-utero environment, but as tobacco use follows a social gradient, this should primarily be seen as a reflection of social position [42–44]. No data were missing *on maternal smoking habits*. We also included other sociodemographic variables associated with school achievement such as parental level of education and family type [45–47]. *Father’s highest education* and *mother’s highest education* was observed the year the child received the final grades of compulsory school (compulsory education/two years of upper secondary education/three years of upper secondary education/university education, two years or more including postgraduate education). Data were missing for 3% of the cohort. *Family type* was categorized as married/cohabiting biological parents when the child was 16 years of age (yes/no). Data were missing for 0.3%. Our cohort consisted of only individuals born in Sweden. However, to control for the potential influence of different cultural and immigrant backgrounds, we included *maternal country of birth* (Sweden, Nordic countries, European countries or countries outside Europe). No data were missing for this variable. Finally, we considered a total *municipal average grade point* variable based on data from the Swedish National Agency for Education. This variable was used to adjust for difference in school quality throughout the country. Data were missing for 3%. The study variables are shown in Table 1.

**Table 1:** The study population and background variables stratified by hospitalization exposures.

	Hospitalization, n (%)						Total, n (%)
	Never	1 night	2–5 nights	6–10 nights	11–50 nights	≥ 51 nights	
All individuals	58 725 (51.0)	15 744 (13.7)	23 878 (20.7)	7538 (6.5)	7980 (6.9)	1331 (1.2)	115 196
Boys	28 228 (24.5)	8394 (7.3)	13 110 (11.4)	4252 (3.7)	4488 (3.9)	668 (0.6)	59 140 (51.3)
Girls	30 497 (26.5)	7350 (6.4)	10 768 (9.3)	3286 (2.9)	3492 (3.0)	663 (0.6)	56 056 (48.7)
Maternal country of birth							
Sweden	51 625 (44.8)	14 011 (12.2)	21 053 (18.3)	6593 (5.7)	7027 (6.1)	1170 (1.0)	101 479 (88.1)
Nordic	2361 (2.0)	566 (0.5)	881 (0.8)	275 (0.2)	310 (0.3)	47 (0.0)	4440 (3.9)
European	1461 (1.3)	371 (0.3)	558 (0.5)	200 (0.2)	187 (0.2)	36 (0.0)	2813 (2.4)
Non-European	3278 (2.8)	796 (0.7)	1386 (1.2)	470 (0.4)	456 (0.4)	78 (0.1)	6464 (5.6)
Maternal smoking habits							
No smoking	46 526 (40.4)	12 038 (10.5)	17 934 (15.6)	5556 (4.8)	5861 (5.1)	983 (0.9)	88 898 (77.2)
1–9 cigarettes/day	7790 (6.8)	2327 (2.0)	3671 (3.2)	1206 (1.0)	1205 (1.0)	204 (0.2)	16 403 (14.2)
≥ 10-cigarettes/day	4409 (3.8)	1379 (1.2)	2273 (2.0)	776 (0.7)	914 (0.8)	144 (0.1)	9895 (8.6)
APGAR score 5 minutes							
7–10	58 533 (50.8)	15 652 (13.6)	23 663 (20.5)	7408 (6.4)	7770 (6.7)	1209 (1.0)	114 235 (99.2)
< 7	192 (0.2)	92 (0.1)	215 (0.2)	130 (0.1)	210 (0.2)	122 (0.1)	961 (0.8)
Birth weight							
Normal	55 895 (48.5)	14 931 (13.0)	22 585 (19.6)	7010 (6.1)	7177 (6.2)	1129 (1.0)	108 727 (94.4)
Low or high for gestational age	2830 (2.5)	813 (0.7)	1293 (1.1)	528 (0.5)	803 (0.7)	202 (0.2)	6469 (5.6)
Father’s highest education							
Compulsory	11 693 (10.2)	3331 (2.9)	5082 (4.4)	1690 (1.5)	1815 (1.6)	300 (0.3)	23 911 (20.8)
Two year secondary	22 830 (19.8)	6385 (5.5)	9969 (8.7)	3110 (2.7)	3329 (2.9)	536 (0.5)	46 159 (40.1)
Three year secondary	6439 (5.6)	1684 (1.5)	2605 (2.3)	767 (0.7)	810 (0.7)	144 (0.1)	12 449 (10.8)
University	16 242 (14.1)	3955 (3.4)	5645 (4.9)	1759 (1.5)	1783 (1.5)	317 (0.3)	29 701 (25.8)
Mother’s highest education							
Compulsory	9015 (7.8)	2698 (2.3)	4328 (3.8)	1406 (1.2)	1518 (1.3)	267 (0.2)	19 232 (16.7)
Two year secondary	24 880 (21.6)	6892 (6.0)	10 522 (9.1)	3379 (2.9)	3535 (3.1)	573 (0.5)	49 781 (43.2)
Three year secondary	6725 (5.8)	1687 (1.5)	2527 (2.2)	751 (0.7)	795 (0.7)	129 (0.1)	12 614 (11.0)
University	17 420 (15.1)	4316 (3.7)	6267 (5.4)	1915 (1.7)	2028 (1.8)	351 (0.3)	32 397 (28.0)
Family type							
Married/cohabiting	47 390 (41.1)	12 447 (10.8)	18 600 (16.1)	5867 (5.1)	6096 (5.3)	1028 (0.9)	91 428 (79.4)
Not married/cohabiting	11 335 (9.8)	3297 (2.9)	5278 (4.6)	1671 (1.5)	1884 (1.6)	303 (0.3)	23 768 (20.6)

### Statistical analyses

We used multiple linear regression to analyze how hospitalizations were associated to overall grade points in the ninth grade of compulsory school. Overall grade points is a continuous and normally distributed variable. The association between hospitalizations and eligibility for upper secondary education was analyzed using logistic regression. Two separate analyzes were run: three models × 2 for each outcome in which covariates were added stepwise using the method “enter”. In the first set of analysis we included the variable number of nights hospitalized years 0–16 differentiated into five categories: 1 night, 2–5 nights, 6–10 nights, 11–50 nights, ≥ 51 nights. This enabled us to examine whether the relationship differed depending on how many nights the child had been hospitalized. In the second set of analysis we examined the timing of a hospitalization and its associated with school achievement. The 11–50 and ≥ 51 nights hospitalized were merged with the category ≥ 6 nights. Interaction terms for gender and hospitalization exposure years 0–16 and for the different age ranges were created and fitted into the models. Individuals not hospitalized served as a reference group in the regression models. Analyzes were performed using SPSS version 24.

## Results

Table 1 presents the distribution of study variables across the study population. In total, 56 471 (49%) individuals had ever been hospitalized from their year of birth until their 16^th^ year of life. Table 2 presents the results of the multiple linear regression analysis using *overall grade points* as the outcome. The first crude model show that the grade points were lower for individuals that had been hospitalized 1 night (β = -7.05, *p* < 0.001), 2–5 nights (β = -10.27, *p* < 0.001), 6–10 nights (β = -14.64, *p* < 0.001), 11–50 nights (β = -19.84, *p* < 0.001) and ≥ 51 nights (β = -30.23, *p* < 0.001) as compared with their healthier counterparts. In the second model, all covariates were added. This attenuated the strength of the associations, however, even after adjustment for sociodemographic background factors and birth health status, the results clearly show that the overall grade points is significantly lower the longer the individual has been hospitalized. The third model, in which interaction terms were entered, reveal a significant interaction between hospitalization and gender: being a girl and having been hospitalized for 1 night (β = -2.13, p < 0.05), 2–5 nights (β = -2.19, p < 0.05), 6–10 nights (β = -2.98, p < 0.05) 11–50 nights (β = 3.82, p < 0.01) or ≥ 51 nights (β = - 10.28, p < 0.01) was significantly associated with lower overall grade points.

**Table 2:** Linear regression models showing associations between hospitalizations and *overall grade points*, unstandardized beta-coefficients, standard error in parentheses.

	Model 1	Model 2	Model 3
Hospitalization age 0–16			
No hospitalizations	Ref.	Ref.	Ref.
1 night	-7.05 (.58)***	-3.45 (.52)***	-2.39 (.72)**
2–5 nights	-10.27 (.50)***	-5.00 (.44)***	-3.94 (.61)***
6–10 nights	-14.64 (.80)***	-8.26 (.72)***	-6.87 (.96)***
11–50 nights	-19.84 (.79)***	-12.82 (.71)***	-11.10 (.96)***
≥ 51 nights	-30.23 (1.99)***	-26.00 (1.79)***	-20.93 (2.54)***
Gender			
Boys		Ref.	Ref.
Girls		22.13 (.34)***	23.42 (.48)***
Maternal country of birth			
Sweden		Ref.	
Nordic		-3.04 (.90)**	-3.07 (.90)**
European		-.50 (1.13)	-.51 (1.13)
Non-European		-1.59 (.78)*	-1.66 (.78)*
Maternal smoking habits			
No smoking		Ref.	Ref.
1–9 cigarettes/day		-11.90 (.50)***	-11.89 (.50)***
≥ 10 cigarettes/day		-20.70 (.64)***	-20.70 (.64)***
Low or high birthweight			
No		Ref.	Ref.
Yes		-2.44 (.75)**	-2.43 (.75)***
APGAR score 5 minutes			
Normal		Ref.	Ref.
Low		-3.07 (1.96)	-3.00 (1.96)
Father’s education			
Compulsory		Ref.	Ref.
Two year secondary		7.86 (.45)***	7.88 (.45)***
Three year secondary		20.11 (.64)***	20.11 (.64)***
University		29.69 (.54)***	29.67 (.54)***
Mother’s education			
Compulsory		Ref.	Ref.
Two year secondary		16.51 (.51)***	16.52 (.51)***
Three year secondary		32.00 (.68)***	31.96 (.68)***
University		39.41 (.59)***	39.37 (.59)***
Family type			
Married/cohabiting		Ref.	Ref.
Not married/cohabiting		-15.66 (.42)***	-15.63 (.42)***
Municipal average			
overall grade points		0.54 (.1)***	0.54 (.1)***
Sex × 1 night			-2.13 (1.04)*
Sex × 2–5 nights			-2.19 (.89)*
Sex × 6–10 nights			-2.98 (1.44)*
Sex × 11–50 nights			-3.82 (1.43)**
Sex × ≥ 51 nights			-10.28 (3.57)**
Constant	211.30 (.26)***	61.18 (4.24)***	60.46 (4.24)***
N	111 765	111 765	111 765
*R*^2^	0.01	0.20	0.20

* = p < 0.05

** = p < 0.01

*** = p < 0.001, *R*^2^ = Adjusted R Square.

Table 3 presents the results of the second analysis using *overall grade points* as the outcome in which we specifically focused on age at illness. The three models in the table, one for each age period, are fully adjusted for all the covariates and clearly show that hospitalizations further up in the ages have greater consequences for the grade points. For all the interaction terms included, we only found significant associations between gender and hospitalization 2–5 nights (β = -8.37, *p* < 0.001) and ≥ 6 nights (β = - 9.57, *p* < 0.001) for ages 13–16.

**Table 3:** Standardized beta-coefficients of *overall grade points* by hospitalization timing and gender interactions. Standard error in parentheses.

	Model 1a	Model 1b	Model 1c
1 night ages 0–6	-1.42 (.78)		
2–5 nights ages 0–6	-2.47 (.65)***		
≥ 6 nights ages 0–6	-5.65 (.80)***		
Gender × 1 night ages 0–6	-1.57 (1.14)		
Gender × 2–5 nights ages 0–6	-1.31 (.97)		
Gender × ≥ 6 nights ages 0–6	-1.68 (1.20)		
1 night ages 7–12		-3.80 (.94)***	
2–5 nights ages 7–12		-5.16 (1.02)***	
≥ 6 nights ages 7–12		-13.87 (1.65)***	
Gender × 1 night ages 7–12		-1.27 (1.42)	
Gender × 2–5 nights ages 7–12		0.52 (1.52)	
Gender × ≥ 6 nights ages 7–12		0.61 (2.47)	
1 night ages 13–16			-8.78 (1.09)***
2–5 nights ages 13–16			-8.61 (1.27)***
≥ 6 nights ages 13–16			-21.66 (1.83)***
Gender × 1 night			-3.04 (1.61)
Gender × 2–5 nights			-8.37 (1.78)***
Gender × ≥ 6 nights			-9.57 (2.49)***
Constant	65.44 (4.54)***	67.31 (4.54)***	68.62 (4.52)***
N	111 765	111 765	111 765
*R*^2^	0.20	0.20	0.21

*** = *p* < 0.001. *R*^2^ = Adjusted R Square. All models are fully adjusted for all the covariates.

Table 4 presents the results of the logistic regression analysis using *ineligibility for upper secondary education* as the outcome. The first unadjusted model show that being exposed to health problems requiring hospitalizations 1 night (OR = 1.25, CI = 1.17–1.33), 2–5 nights (OR = 1.34, CI = 1.27–1.42), 6–10 nights (OR = 1.51, CI = 1.76–2.03), 11–50 nights (OR = 1.89, CI = 1.76–2.03) or ≥ 51 nights (OR = 2.66, CI = 2.27–3.13) significantly increased the odds of ineligibility for upper secondary education: the longer hospital stay, the higher the odds for ineligibility. The second model show that, even after adjustment for all the covariates, hospitalization 1 night (OR = 1.17, CI = 1.10–1.25), 2–5 nights (OR = 1.21, CI = 1.15–1.28), 6–10 nights (OR = 1.33, CI = 1.22–1.44), 11–50 nights (OR = 1.66, CI = 1.54–1.79) or ≥ 51 nights (OR = 2.48, CI = 2.10–2.94) significantly increased the odds of ineligibility for upper secondary education (*p* < 0.001). The third model was adjusted for the interaction terms and revealed a non-significant interaction between hospitalizations and gender, except for hospitalization 11–50 nights (*p* < 0.05).

**Table 4:** Hospitalizations and odds ratios (OR) of *ineligibility for upper secondary education*. Confidence intervals (CI) in parentheses.

	Model 1	Model 2	Model 3
Hospitalization age 0–16			
No hospitalization	1	1	1
1 night	1.25 (1.17–1.33)***	1.17 (1.10–1.25)***	1.16 (1.07–1.27)***
2–5 nights	1.34 (1.27–1.42)***	1.21 (1.15–1.28)***	1.19 (1.10–1.27)***
6–10 nights	1.51 (1.27–1.42)***	1.33 (1.22–1.44)***	1.24 (1.11–1.38)***
11–50 nights	1.89 (1.76–2.03)***	1.66 (1.54–1.79)***	1.52 (1.38–1.69)***
≥ 51 nights	2.66 (2.27–3.13)***	2.48 (2.10–2.94)***	2.43 (1.93–3.05)***
Gender			
Boys		1	1
Girls		0.80 (.77-.84)***	0.77 (0.73–0.82)***
Maternal country of birth			
Sweden		1	1
Nordic		1.14 (1.03–1.26)**	1.15 (1.04–1.27)**
European		1.30 (1.03–1.26)***	1.30 (1.14–1.49)***
Non-European		1.29 (1.19–1.40)***	1.30 (1.19–1.41)***
Maternal smoking habits			
No smoking		1	1
1–9 cigarettes/day		1.44 (1.37–1.52)***	1.44 (1.37–1.52)***
≥ 10 cigarettes/day		1.71 (1.61–1.82)***	1.71 (1.60–1.82)***
Low or high birthweight			
No		1	1
Yes		1.17 (1.29–1.37)***	1.17 (1.07–1.27)***
APGAR score 5 minutes			
Normal		1	1
Low		1.09 (0.87–1.37)	1.08 (0.86–1.36)
Father’s education			
Compulsory		1	1
Two year secondary		0.73 (0.69–0.76)***	0.73 (0.69–0.76)***
Three year secondary		0.49 (0.45–0.54)***	0.49 (0.45–0.54)***
University		0.38 (0.35–0.41)***	0.38 (0.35–0.41)***
Mother’s education			
Compulsory		1	1
Two year secondary		0.59 (0.56–0.62)***	0.59 (0.56–0.62)***
Three year secondary		0.32 (0.30–0.36)***	0.33 (0.30–0.36)***
University		0.32 (0.29–0.34)***	0.32 (0.30–0.35)***
Family type			
Married/cohabiting		1	1
Not married/cohabiting		1.58 (1.51–1.66)***	1.58 (1.50–1.65)***
Municipal average		0.98 (0.98–0.98)***	0.98 (0.98–0.98)***
overall grade points			
Sex × 1 night			1.00 (0.88–1.47)
Sex × 2–5 nights			1.04 (0.93–1.16)
Sex × 6–10 nights			1.17 (0.99–1.38)
Sex × 11–50 nights			1.21 (1.04–1.41)*
Sex × ≥ 51 nights			1.04 (0.74–1.46)
Constant	-2.48 (.01)***	1.88 (.27)***	1.90 (.27)***
N	111 765	111 765	111 765
*R*^2^	0.009	0.11	0.11

* = p < 0.05

** = p < 0.01

*** = p < 0.001, *R*^2^ = Nagelkerke R Square.

Table 5 presents the results of the second analysis using *ineligibility for upper secondary education* as the outcome, in which we specifically focused on age at illness. The three models in the table, one for each age period, are fully adjusted for all the covariates and show that hospitalizations further up in the ages are associated with higher odds ratios (OR) of ineligibility for upper secondary education. For all the interaction terms included, we only found significant associations for gender and hospitalization ≥ 6 nights for ages 0–6 (*p* < 0.01). Overall, the results suggested that if we consider school achievement in the three core subjects and ineligibility for secondary education, gender does not play a part. However, when considering school achievement across all school subjects as overall grade points, girls with health problems are associated with lower school achievements than boys with health problems.

**Table 5:** Hospitalization timing, gender interactions and odds ratios (OR) for *ineligibility for upper secondary education*. Confidence intervals in parentheses.

	Model 1a	Model 1b	Model 1c
1 night ages 0–6	1.06 (0.97–1.16)		
2–5 nights ages 0–6	1.12 (1.04–1.21)**		
≥ 6 nights ages 0–6	1.21 (1.11–1.32)***		
Gender × 1 night ages 0–6	1.02 (0.88–1.16)		
Gender × 2–5 nights ages 0–6	1.03 (0.92–1.16)		
Gender × ≥ 6 nights ages 0–6	1.22 (1.07–1.39)**		
1 night ages 7–12		1.21 (1.09–1.34)***	
2–5 nights ages 7–12		1.22 (1.09–1.37)***	
≥ 6 nights ages 0–6		1.55 (1.31–1.82)***	
Gender × 1 night ages 7–12		0.97 (0.82–1.14)	
Gender × 2–5 nights ages 7–12		0.89 (0.75–1.07)	
Gender × ≥ 6 nights ages 7–12		1.03 (0.90–1.33)	
1 night ages 13–16			1.34 (1.19–1.51)***
2–5 nights ages 13–16			1.49 (1.31–1.70)***
≥ 6 nights ages 13–16			2.10 (1.77–2.49)***
Gender × 1 night ages 0–6			1.11 (0.93–1.33)
Gender × 2–5 nights ages 7–12			1.03 (0.86–1.25)
Gender × ≥ 6 nights ages 13–16			1.09 (0.86–1.38)
Constant	1.81 (.25)***	1.74 (.25)***	1.69 (.25)***
N	111 765	111 765	111 765
*R*^2^	0.10	0.11	0.11

** = *p* < 0.01

*** = *p* < 0.001. *R*^2^ = Nagelkerke R Square. All models are fully adjusted for all the covariates.

## Discussion

This study found that children with health problems were less successful in school. Even with the crude measure of health problems that we used in our study, i.e., hospitalizations, we discerned that Swedish children, in rather contemporary conditions, displayed lower school achievements as compared to their healthier counterparts. Consequently, health is a significant contributor to inequalities in education.

We found that girls who had been hospitalized in general had poorer achievements than boys, which raises several questions. The school performance difference can be explained and understood through norms and ideals, the social gender role constructs that manifest in schools and classrooms, which lead to teachers treating boys and girls differently [21]. Similar gender-driven mechanisms might exist in the case of health problems affecting girls and boys differently in their schooling. Examples from Sweden have shown that girls and boys differ in the problems they seek medical care for [48], how they cope with school-related stress [49] and how they perceive demands and appraisals in the school environment [50]. Despite possible explanations in the existing literature, we cannot within the framework of this study fully explain the gender differences detected in our findings, and hence they require further empirical analysis.

Regarding our third aim—to investigate how the timing of a health problem affects later school achievement—we learned that individuals who were exposed to hospitalizations closer to the actual grading suffered a poorer outcome. Thus, there seems to be a timing effect. In our study, junior high school was found to be a critical period regarding the effect on compulsory school grades of exposure to health problems requiring hospitalization. Although the years prior to formal schooling are important concerning school readiness [51], it seems easier to recuperate from diseases during the infant years. However, when already in school, especially close to actual grading, disruptions like exposure to health problems have a greater negative impact on school achievement. Good health can be seen as a buffer against life stressors [52] and junior high school is a period often associated with various life stressors, educational progress and transition into adolescence. Thus, this period is understandably critical.

Limitations of the study should be mentioned. Hospitalizations as a measure captures only general non-specific health problems. Detailed information from the NPR, such as diagnostic type, would allow us to access specifics of a health problem, especially when trying to find reasons for the observed gender differences, which is imperative. Grading in compulsory school only occurred in grade 9 for this cohort, outcome measures at several occasions would have improved the study. Moreover, other individuals who suffer from other disabilities/impairments but who did not receive similar care (i.e. requiring hospitalization) and who we could not account for may exist in our sample. Further, the age groupings 0–6, 7–12 and 13–16 describe different lengths of time and thus their coefficients are not directly comparable.

The main strengths of our study are the high quality datasets; the multiple sources of linked data, in particular access to the MBR which allowed us to control for health selection that might occur at birth. This enabled us to observe a whole cohort of individuals from prior to birth until graduation from compulsory school. In addition, the study used two different educational achievement variables, where overall grade points indicate differences in levels of school achievement while eligibility for continuation to upper secondary education indicate problems of completing education at all.

In conclusion, this study found that health problems, measured as hospitalizations, was significantly association with lower school achievements among Swedish children. Girls exposed to hospitalizations had in general poorer school achievements compared to boys. Support services should pay particular attention to the needs of young people when they suffer health problems that lead to hospitalizations during junior high school as this seemingly is a critical period in relation to their final achievement at compulsory school.

## References

[1] Skolverket [Swedish National Agency for Education]. What influences educational achievement in Swedish Schools? A systematic review and summary analysis. Stockholm: Skolverket; 2009.

[2] Hattie JA. Visible learning A synthesis of over 800 meta-analyses relating to achievement. New York: Routledge; 2009.

[3] Basch CE. Healthier students are better learners: a missing link in school reforms to close the achievement gap. Journal of School Health. 2011;81:593–598. 10.1111/j.1746-1561.2011.00632.x 21923870 10.1111/j.1746-1561.2011.00632.x

[4] Bhutta A, Cleves MA, Casey PH, Anand K. Cognitive and behavioral outcomes of school-aged children who were born preterm: A meta analysis. JAMA, The Journal of the American Medical Association. 2002;288(6):728–737. 12169077 10.1001/jama.288.6.728

[5] Stjernqvist K, Svenningsen N. Ten‐year follow‐up of children born before 29 gestational weeks: health, cognitive development, behaviour and school achievement. Acta Paediatrica. 1999;88(5):557–562. 10426181 10.1080/08035259950169594

[6] McDougall J, King G, de Wit DJ, Miller LT, Hong S, Offord DR. et al Chronic physical health conditions and disability among Canadian school-aged children: A national profile. Disability and Rehabilitation: An International, Multidisciplinary Journal. 2004;26(1):35–45. 10.1080/09638280410001645076 14660197

[7] Currie J, Stabile M, Manivong P, Roos LL. Child health and young adult outcomes. Journal of Human Resources. 2010;45(3):517–548.

[8] Shaw SR, McCabe PC. Hospital-to-school transition for children with chronic illness: meeting the new challenges of an evolving health care system. Psychology in the Schools. 2007;45(1):74–87.

[9] Moonie S, Sterling DA, Figgs LW, Castro M. The relationship between school absence, academic performance, and asthma status. Journal of School Health. 2008;78:140–148. 10.1111/j.1746-1561.2007.00276.x 18307609 10.1111/j.1746-1561.2007.00276.x

[10] Collett BR, Wehby GL, Barron S, Romitti PA, Ansley TN, Speltz ML. Academic achievement in children with oral clefts versus unaffected siblings. Journal of Pediatric Psychology. 2014;39(7):743–751. 10.1093/jpepsy/jsu049 24993102 10.1093/jpepsy/jsu049 PMC4107579

[11] Needham BL, Crosnoe R, Muller C. Academic failure in secondary school: the inter-related role of health problems and educational context. Social Problems. 2004;51(4):569–586. 20354573 10.1525/sp.2004.51.4.569 PMC2846654

[12] Suhrcke M, de Paz Nieves C. The impact of health and health behaviours on educational outcomes in high income countries: a review of the evidence. Copenhagen: WHO Regional Office for Europe; 2011.

[13] Eriksson C. Kunskap om hälsa och lärande–en översikt av ett forskningsfält under utveckling [Knowledge of health and learning–an overview of a research field under development]. Stockholm: Vetenskapsrådet, Lilla Rapportserien 2012:2.

[14] Mörk E, Sjögren A, Svaleryd H. Hellre rik och frisk–om familjebakgrund och barns hälsa [Rather rich and healthy–about family background and children’s health] The Institute for Evaluation of Labour Market and Education Policy (IFAU). Uppsala: Rapport 2015:13.

[15] Voyer D, Voyer SD. Gender differences in scholastic achievement: a meta-analysis. Psychological Bulletin. 2014;140(4):1174–1204. 10.1037/a0036620 24773502 10.1037/a0036620

[16] Torsheim T, Ravens-Sieberer U, Hetland J, Välimaa R, Danielson M, Overpeck M. Cross-national variation of gender differences in adolescent subjective health in Europe and North America. Social Science & Medicine. 2006;62(4):815–27. 16098649 10.1016/j.socscimed.2005.06.047

[17] Hagquist C. Psychosomatic health problems among adolescents in Sweden–are time trends gender related? European Journal of Public Health. 2009;19:331–336. 10.1093/eurpub/ckp031 19304732 10.1093/eurpub/ckp031

[18] The Public Health Agency of Sweden. Health behavior in school-aged children (HBSC), results from Sweden of the 2013/14 WHO study. 2014.

[19] Holmberg LI, Hellberg D. Age-related gender differences of relevance for health in Swedish adolescents. International Journal of Adolescent Medicine And Health. 2007;19(4):447–457. 18348420 10.1515/ijamh.2007.19.4.447

[20] Stenmark H, Bergström E, Hägglöf B, Öhman A, Petersen S. Mental problems and their socio-demographic determinants in young schoolchildren in Sweden, a country with high gender and income equality, Scandinavian Journal of Public Health. 2015;44(1):18–26. 10.1177/1403494815603544 26392422 10.1177/1403494815603544

[21] Wernersson I. SOU 2010:51 [Government official reports 2010:51]. Könsskillnader i skolprestationer–Idéer om orsaker [Gender differences in school performance–Ideas about the causes]. Stockholm: Utbildningsdepartementet [Ministry of education].

[22] Öhrn E, Holm A-S. Att lyckas i skolan. Om skolprestationer och kön i olika undervisningspraktiker [To succeed at school. About school achievements and gender in different teaching practices] Gothenburg Studies in Educational Sciences, 363 Göteborg: Acta Universitatis Gothoburgensis; 2014.

[23] Aanesen F, Meland E, Torp S. Gender differences in subjective health complaints in adolescence: The roles of self-esteem, stress from schoolwork and body dissatisfaction. Scandinavian Journal of Public Health, 2017;(45)4, 389–396. 10.1177/1403494817690940 28385116

[24] Savoye I, Moreau N, Brault MC, Levêque A, Godin I. Well-being, gender, and psychological health in school-aged children. Archives of Public Health. 2015;21;73:52. 10.1186/s13690-015-0104-x PMC4685636 26693278

[25] Case A, Fertig A, Paxson C. The lasting impact of childhood health and circumstance. Journal of Health Economics. 2005;24(2):365–389. 10.1016/j.jhealeco.2004.09.008 15721050 10.1016/j.jhealeco.2004.09.008

[26] Palloni A. Reproducing inequalities: luck wallets, and the enduring effects of childhood health. Demography. 2006;43:587 17236536 10.1353/dem.2006.0036

[27] Jackson MI. Understanding links between adolescent health and educational attainment. Demography. 2009;46(4):671–694. 20084824 10.1353/dem.0.0078 PMC2831357

[28] Jackson MI. A life course perspective on child health, cognition and occupational skill qualifications in adulthood: evidence from a British cohort. Social Forces. 2010;89(1):89–116. 10.1353/sof.2010.0101 PMC3893027 24443594

[29] Wodrich DL, Hasan K, Parent KB. Type 1 diabetes mellitus and school: a review. Pediatric Diabetes. 2011;12:63–70. 10.1111/j.1399-5448.2010.00654.x 20546162 10.1111/j.1399-5448.2010.00654.x

[30] Dahlquist G, Källén B, (On behalf of the Swedish Childhood Diabetes Study Group). School performance in children with type I diabetes–a population-based register study. Diabetologia. 2007;50:957–964. 10.1007/s00125-007-0615-2 17333107 10.1007/s00125-007-0615-2

[31] Milton B, Holland P, Whitehead M. The social and economic consequences of childhood-onset type 1 diabetes mellitus across the lifecourse: a systematic review. Diabetic Medicine. 2006;23:821–829. 10.1111/j.1464-5491.2006.01796.x 16911617 10.1111/j.1464-5491.2006.01796.x

[32] Hannonen R, Komulainen J, Riikonen R, Ahonen T, Eklund K, Tolvanen, et al Academic skills in children with early-onset type 1 diabetes: the effects of diabetes-related risk factors. Developmental Medicine & Child Neurology. 2012;54(5):457–463. 22590723 10.1111/j.1469-8749.2012.04248.x

[33] Persson S, Dahlquist G, Gerdtham UG, Steen Carlsson K. Impact of childhood-onset type 1 diabetes on schooling: population-based register study. Diabetologia. 2013;56:1254 10.1007/s00125-013-2870-8 23435847 10.1007/s00125-013-2870-8

[34] Lindgren U, Nilsson K, de Luna X, Ivarsson A. Data resource profile: Swedish microdata research from childhood into lifelong health and welfare (Umeå SIMSAM Lab). International Journal of Epidemiology. 2016;45(4):1075–1075. 10.1093/ije/dyv358 27170765 10.1093/ije/dyv358

[35] Källén B., & Källén K. (2003). *The Swedish Medical Birth Register*: *A summary of content and quality*. Socialstyrelsen [Swedish National Board of Health and Welfare].

[36] Axelsson O. (2003). The Swedish Medical Birth Register. *Acta Obstetricia et Gynecologica Scandinavica*, 83: 491–492. 10.1034/j.1600-0412.2003.00172.x 12780418

[37] Odlind V., Haglund B., Pakkanen M. & Otterblad Olausson P. (2003). Deliveries, mothers and newborn infants in Sweden, 1973–2000: Trends in obstetrics as reported to the Swedish Medical Birth Register. *Acta Obstetricia et Gynecologica Scandinavica*, 86(2): 516–28. 12780422

[38] Ludvigsson J. F., Andersson E., Ekbom A., Feychting M., Kim J.-L., Reuterwall C., Olausson P. O. (2011). External review and validation of the Swedish National Inpatient Register. *BMC Public Health*, 11: 450 10.1186/1471-2458-11-450 21658213 10.1186/1471-2458-11-450 PMC3142234

[39] Grönhagen C., Nilzén K., Seifert O., & Throslund K. (2017). Bullous Pemphigoid: Validation of the National Patient Register in two counties in Sweden, 2001 to 2012, *Acta Dermato-Venereologica*, 97: 32–35. 10.2340/00015555-2456 27171523 10.2340/00015555-2456

[40] Stuart A, Otterblad Olausson P, Källen K. Apgar scores at 5 minutes after birth in relation to school performance at 16 years of age. Obstetrics & Gynecology. 2011;118(2):201–208. 21734618 10.1097/AOG.0b013e31822200eb

[41] Torche F, Echevarría G. The effect of birthweight on childhood cognitive development in a middle-income country. International Journal of Epidemiology. 2011;40: 1008–1018. 10.1093/ije/dyr030 21362701 10.1093/ije/dyr030

[42] Shohaimi S, Luben R, Wareham N, Day N, Bingham S, Welch A, et al Residential area deprivation predicts smoking habit independently of individual educational level and occupational social class. A cross sectional study in the Norfolk cohort of the European Investigation into Cancer (EPIC-Norfolk). Journal of Epidemiological Community Health. 2003;57:270–276. 10.1136/jech.57.4.270 PMC1732421 12646543

[43] Stewart MJ, Brosky G, Gillis A, Jackson S, Johnston G, Kirkland, et al Disadvantaged women and smoking. Canadian Journal of Public Health. 1996;87:257–260. 8870305

[44] Osler M, Holstein B, Avlund K, Rasmussen NK. Socioeconomic position and smoking behaviour in Danish adults. Scandinavian Journal of Public Health. 2001;29:32–39. 11355714

[45] Woessman L. An international look at the single-parent: family structure matters more for U.S. students. Education Next. 2015;15(23):42–49.

[46] Björklund A, Lindahl M, Sund K. Family background and school performance during a turbulent era of school reforms. Swedish Economic Policy Review. 2003;10:111–136.

[47] Davis-Kean PE. The influence of parent education and family income on child achievement: The indirect role of parental expectations and the home environment. Journal of Family Psychology. 2005;19:294–304. 10.1037/0893-3200.19.2.294 15982107 10.1037/0893-3200.19.2.294

[48] Clausson EK, Köhler L, Berg A. Schoolchildren’s health as judged by Swedish school nurses—a national survey. Scandinavian Journal of Public Health, 2008;36:690–697. 10.1177/1403494808090671 18684783 10.1177/1403494808090671

[49] Wilhsson M, Svedberg P, Högdin S, Nygren JM. Strategies of adolescent girls and boys for coping with school-related stress. Journal of School Nursing. 2017;33(5):374–382. 10.1177/1059840516676875 28891410 10.1177/1059840516676875

[50] Låftman SB, Modin B. School-performance indicators and subjective health complaints: are there gender differences? Sociology of Health & Illness. 2012;34(4): 608–625. 22103817 10.1111/j.1467-9566.2011.01395.x

[51] Kull M. Early physical health problems as developmental liabilities for school readiness: Associations with early learning contexts and family socioeconomic status. Doctoral Dissertation. Boston College. 2015. Available from: http://hdl.handle.net/2345/bc-ir:104143.

[52] Forrest CB, Bevans KB, Riley AW, Crespo R, Louis TA. School outcomes of children with special health care needs. Pediatrics. 2011;128(2):303–312. 10.1542/peds.2010-3347 21788226 10.1542/peds.2010-3347 PMC3387854

